# Effect of web-based health education on nursing students’ knowledge, adaptive healthy measures and attitudes regarding polycystic ovary syndrome: a randomized controlled trial

**DOI:** 10.1186/s12912-024-02015-7

**Published:** 2024-07-15

**Authors:** Rasha A. Mohamed, Nagwa N. Taref, Nehmedo E. Osman, Nawal Hamdy Ahmed Keshta, Mahmoud A. Alboghdady, Marzouk M. Marzouk, Abeer A. Almowafy, Eman A. Fadel

**Affiliations:** 1 Community Health Nursing, Faculty of Nursing, Mansoura University, Mansoura, Egypt; 2 College of Applied Medical Sciences, University of Bisha, Bisha, Saudi Arabia; 3 Woman’s Health and Midwifery Nursing, Faculty of Nursing, Mansoura University, Mansoura, Egypt; 4 Department of Obstetrics and Gynecology, Faculty of Medicine, Girls Branch, Al Azhar University, Cairo, Egypt; 5 Obstetrics and gynecology at ART unit, International Islamic institute Al-Azhar University, Cairo, Egypt; 6 Public Health and Community Medicine, Faculty of Medicine, Al-Azhar University, Damietta, Egypt; 7 International Islamic Center for Population Studies and Research, Al-Azhar University, Cairo, Egypt

**Keywords:** Polycystic ovary syndrome, Knowledge, Adaptive healthy measures, Attitudes, Web-based learning

## Abstract

**Background:**

Polycystic ovary syndrome (PCOS) is a complex endocrine disorder affecting women of reproductive age, and it has emerged as a significant global public health issue. This study aimed to investigate the effects of web-based health education on nursing students’ knowledge, adaptive healthy measures, and attitudes toward PCOS.

**Methods:**

A two-group randomized controlled trial (RCT) with pre-test and immediate post-test assessments was conducted. Study participants were recruited using a simple random sampling method from the Faculty of Nursing, Mansoura University, Egypt. A questionnaire consisting of six sections was developed to collect data, which was analyzed with the SPSS 23.0 using Student’s t-test, Pearson’s correlation test, and chi-square test analysis of variance.

**Results:**

The analysis revealed a significant increase in knowledge scores post-intervention, with the web-based learning groups (32.2 ± 10.5) outperforming the traditional learning group (22.1 ± 10.2), with (*p* < 0.05). Similarly, there was a notable improvement in adaptive healthy measures scores post-intervention, with the web-based learning group (8.9 ± 2.4) showing better results than the traditional group (6.5 ± 2.9), with (*p* < 0.05). In terms of attitudes toward PCOS, the web-based group (18.2 ± 4.9) displayed a significant improvement compared to the traditional group (11.7 ± 5.2), with (*p* < 0.05).

**Conclusions:**

The findings suggest that web-based learning is more effective than traditional methods in enhancing nursing students’ knowledge, adaptive healthy measures, and attitudes toward PCOS.

**Trial Registration:**

: This study was registered by Clinical Trials.gov Identifier: (NCT06192381||https://www.clinicaltrials.gov/) on 5-1-2024.

**Supplementary Information:**

The online version contains supplementary material available at 10.1186/s12912-024-02015-7.

## Key points

PCOS is a significant global community health concern among reproductive-aged women due to hormonal disorders, making community health nurses essential in promoting health and preventing disease through health education.

Web-based health education is seen as a crucial tool in fostering knowledge, promoting adaptive healthy measures, and shaping attitudes in nursing students’ education.

Through web-based health education interventions, students’ understanding of PCOS, adoption of healthy measures, and attitudes are shown to improve compared to traditional methods.

**Supplementary Information:**

The online version contains supplementary material available at 10.1186/s12912-024-02015-7.

## Introduction

Polycystic ovary syndrome (PCOS) is a global health concern that is considered a complex, multi-faceted, and heterogeneous endocrine disorder. It affects 6–20% of women of reproductive age worldwide [1]. According to statistics, one in ten women will experience PCOS before menopause and will have to deal with its implications [2]. In Egypt, teenage girls were found to have PCOS in 6.6% of cases and 12.6% of high-risk cases [3].

According to the Rotterdam consensus, PCOS is defined by the presence of two of three of the following criteria: oligo-amenorrhea, hyperandrogenism, and objective evidence by ultrasound of polycystic ovaries [4]. It is important to note that PCOS increases the risk of other issues such as cardiovascular disorders [5, 6], type 2 diabetes mellitus, depression, dysfunctional ovaries [7], infertility, endometrial carcinoma, and excess levels of male hormones [8]. After recommending specific lifestyle changes and providing supplemental advice, the standard course of treatment for this issue involves symptomatic therapy using a variety of medications, including contraceptives, oral antidiabetics, or antiandrogens [9].

To grow and develop good health, adolescents need access to information, opportunities for life skills development, acceptable, equitable, appropriate, and effective community health services, as well as safe and supportive environments [10]. Universities and colleges are recognized as places to promote the development of lifelong healthy habits, and research on health education in higher education is expanding both nationally and worldwide [11, 12]. Additionally, an individual’s ability to impact their future health depends on their engagement in healthy behaviors while in college. Therefore, when it comes to health education interventions, university students should be the priority [13]. Recently, teenagers have been utilizing the Internet to access health information, particularly in private [14].

E-learning is a convenient way of learning, offering a rich and flexible learning environment. It provides ready access to knowledge relevant to nursing practice and allows for interactions with facilitators and others [15]. Web-based health education offers access to information and improves care coordination. It is independent, acceptable, affordable, and has easily updated contents [16, 17]. Additionally, digitalized health education resources have the potential to increase health literacy and contribute significantly to illness prevention [18].

Health literacy through health education has an impact on community, family, and individual health and has significant potential to help achieve specific Sustainable Development Goals (SDG; SDG 3) [19]. Community health nurses play a crucial role in community health education by promoting health processes and preventing disease within the community [20].

According to studies, there is a knowledge gap among students regarding PCOS, and lifestyle choices may be a risk factor for PCOS [21]. Additionally, unsatisfactory medical consultation experiences that do not fill in their knowledge gaps may cause women to have unmet information needs and hinder their involvement with PCOS treatment and management in the future [22].

Although there is a lack of robust evidence to support the effectiveness of web-based interventions, it appears to be a promising approach to support behavioral health changes through health promotion programs [23, 24], which is increasingly attractive to researchers [25]. Similarly, it is necessary to utilize new educational methods and evaluate their effectiveness in addressing this issue. Therefore, it is important to provide valid information about PCOS through preferred information channels for university students. To understand and identify gaps in knowledge related to this health problem, the researchers of the current study felt the need to conduct this study among university nursing students to increase their awareness, promote adaptive healthy measures, and foster positive attitudes to prevent PCOS-related clinical implications. This study aimed to investigate the effects of a web-based health education on nursing students’ knowledge, adaptive healthy measures, and attitudes toward PCOS. It was hypothesized that implementing web-based health education on PCOS would be more effective in improving nursing students’ knowledge, promoting adaptive healthy measures, and shaping positive attitudes compared to traditional health education.

## Methods

### Study design

**Fig. 1:**
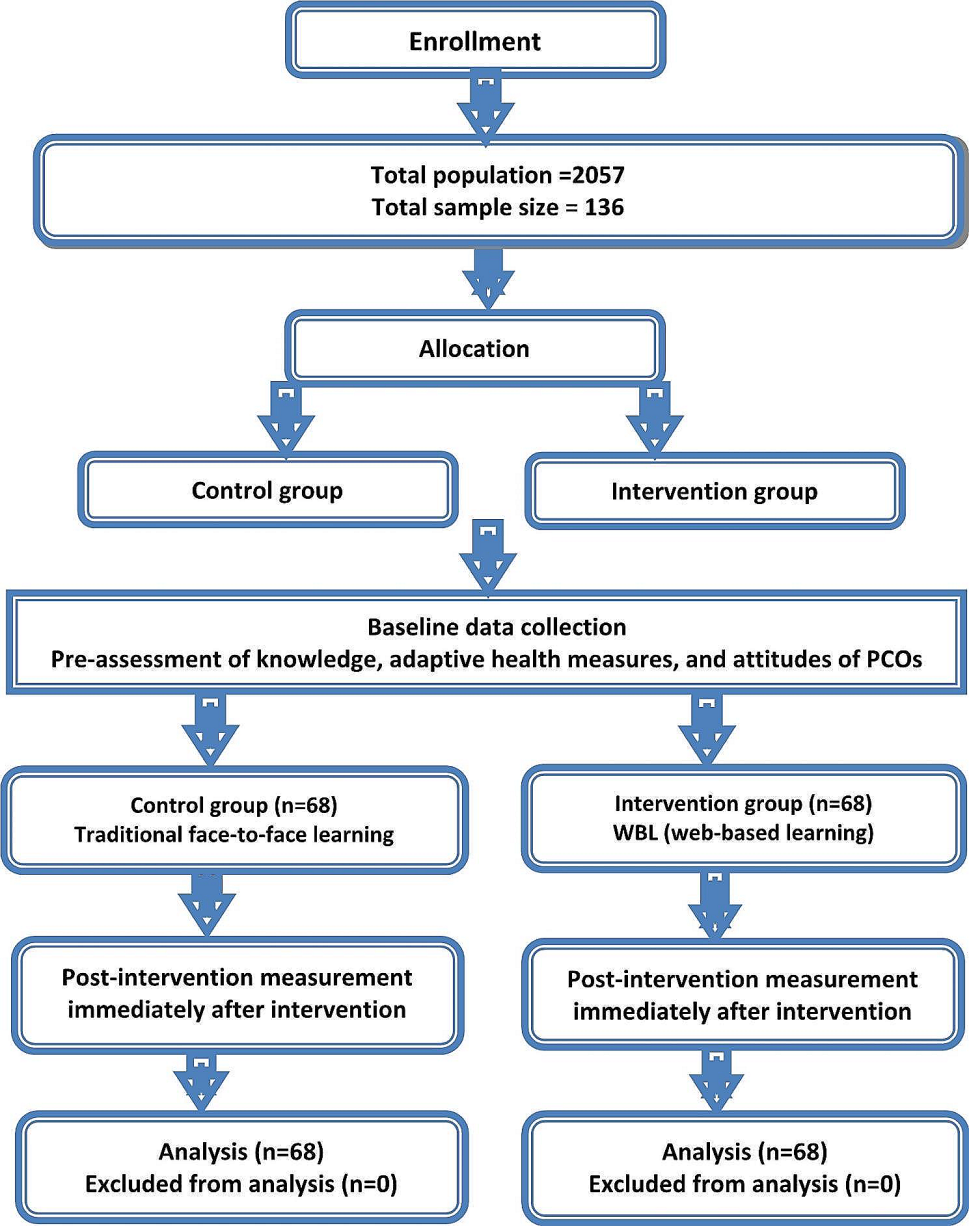
A CONSORT-Style flow chart of participants throughout phases of the study

A randomized controlled trial (RCT) with a pre-test and immediate post-test was utilized for this study. This design was chosen due to its suitability for the existing research hypothesis. The study design and procedures adhered to the criteria outlined in the CONSORT 2010 checklist of information to include when reporting a randomized trial. The flow of the study is illustrated in Fig. 1.

### Study setting

The study took place at the Faculty of Nursing, Mansoura University, Egypt, from December 2022 to May 2023.

### Study sampling and subjects

A simple random sampling technique was used to recruit participants for this study. The target population consisted of nursing students at all academic levels enrolled in a bachelor-level program at the Faculty of Nursing. The inclusion criteria for the study included female university undergraduate nursing students without significant co-morbidities who were willing to provide self-reported data at two assessment points. The exclusion criteria included nursing students with PCOS already receiving treatment, as well as non-consenting participants.

### Sample size

Based on data from the literature [26], considering a level of significance of 5%, and a power of study of 80%, the sample size was calculated using the following formula: n= [(Z_α/2_+Z_β_)^2^×{2(SD)^2^}]/(mean difference between the two groups)^2^ where SD = standard deviation-Z_α/2_: This depends on the level of significance, for 5% this is 1.96 Z_β_: This depends on power, for 80% this is 0.84. Therefore, n=[(1.96 + 0.84)^2^×{2(0.87)^2^}]/(0.42)^2^=67.3 Based on the above formula, the sample size required per group was 68.

### Recruitment and randomization

To ensure a consistent starting point, participants were recruited through the faculty website and academic counseling services. After the initial assessment, participants were randomly assigned to a 1:1 ratio to two groups: (1) the traditional health education group (control group) (*n* = 68) who received health education through the traditional face-to-face methods and (2) the web-based health education group (intervention group) (*n* = 68) who received health education through the Web-Based Learning (WBL). The primary investigator assigned numbers and randomly selected participants using a simple random table to ensure an equal chance of allocation to either group. The researchers employed cluster randomization to reduce contamination bias between the intervention and control groups, ensuring that all patients at the same level received an identical intervention. Patients and researchers were aware of group allocation, but outcome assessors and data analysts were blinded.

### Outcomes

#### Primary outcome

The primary outcomes were improvements in students’ knowledge, adaptive healthy measures, and attitudes toward PCOS. These primary outcomes were measured using the study questionnaire (Parts 3–4 and 5) both before and immediately after the intervention.

#### Secondary outcome

The secondary outcome was students’ satisfaction with the web-based educational sessions, which was measured using the study questionnaire (Part 6) immediately post-intervention.

### Measurement tools

Data was collected using a structured self-administered questionnaire written in Arabic, consisting of six parts.

#### Part 1

Socio-demographic data was gathered to extract information such as age, academic year, residency, and income.

#### Part 2

Students’ medical health assessment data included important present and past medical health histories, as well as family history of PCOS, age of menarche, menstrual amount and regularity, weight, height, body mass index (BMI), and source of information about PCOS.

#### Part 3

Self-administered structured knowledge assessment data about PCOS and its preventive measures was utilized both before and after the implementation of the web-based health education regarding PCOS. The questionnaire was constructed by the researchers based on data retrieved from sources [27, 28] and through expert consensus. It was classified into eight main categories related to the definition, number, and functions of ovaries (5 marks), the definition of PCOS (1 mark), etiology (4 marks), risk factors (4 marks), signs and symptoms (12 marks), diagnosis (4 marks), complications (11 marks), and preventive measures and treatment of PCOS (18 marks). Each accurate response received one mark, while incorrect responses received a score of zero. The scores of the items in each area of knowledge were summed up and divided by the number of items, giving a mean score for each area. The total items of knowledge were 59 resulting in a total of 59 marks. These scores were converted into a percent score. Three levels of knowledge were distinguished: poor level (less than 50% of the possible points, good level (more than 75% of total scores), and fair level (50–75% of total scores). A greater score indicated a better participant understanding of PCOS. The tool’s reliability was assessed using the test-retest and internal consistency for all items. The calculated Cronbach’s alpha for the tool was 0.81, which indicates an acceptable reliability.

#### Part 4

Self-reported structured data on adaptive healthy measures related to PCOS was utilized before and after the implementation of the intervention. This data was created by the researchers using information from [27, 28] and input from experts. It was divided into three main categories: diet, weight reduction, and maintaining a healthy lifestyle. The total score for adaptive healthy measures ranged from 0 to 14 points. The level of adaptive healthy measures for PCOS was split into two categories: satisfactory adaptive healthy measures, which included scores of 60% of total possible points (8.4 points and above), and unsatisfactory adaptive healthy measures, which included scores less than 60% of the total possible points (less than 8.4 points). A higher score indicated better adaptive healthy measures for participants with PCOS. The tool’s reliability was assessed using the test–retest and internal consistency for all items. The calculated Cronbach’s alpha for the tool was 0.79, which indicates an acceptable reliability.

#### Part 5

Self-administered structured attitudes scale. It was constructed by the researchers based on data retrieved from sources [27, 28]. It consisted of 14 statements requiring a response on a three-point Likert rating scale (agree, neutral, and disagree). In the category of positive attitudes, agree responses received two points, neutral responses received one point, and disagree responses received zero points. In the Statistical Package for Social Science (SPSS), the scoring method for negative attitudes was flipped, with zero points awarded for agreeing, one point for being neutral, and two points for disagreeing. The scale’s reliability was assessed using the test–retest and internal consistency for all items. The calculated Cronbach’s alpha for the tool was 0.77, which indicates an acceptable reliability.

#### Part 6

Students’ feedback on the designed web-based health education self-administered scale. The scale was adapted from [29, 30]. This scale was used to assess the educational module’s usefulness, usability, objectives, learning materials, design, and structure. It was composed of 26 criteria and was classified into six domains. The scale required a response on a four-point Likert rating scale that ranged from one to four: the usefulness of the module (4 items); objectives and learning materials (7 items); the design and structure (4 items). In addition to, interaction and assessment (3 items); web-based module vs. face-to-face class (4 items); usability and accessibility (4 items). In the scoring system, the total students’ responses were summarized in the form of a weighted (total) mean. An interval of means was created to give interpretations for the weighted mean, and the mean was converted to percent by multiplying the value of the weighted mean by 25. Cronbach’s α coefficient was found to be 0.83 in the present study, compared to 0.86 in the thesis of Ibrahim Ibrahim [30].

### Study questionnaire validation and pilot testing

A jury of five experts in the fields of “Community Health Nursing, Women’s Health, and Midwifery Nursing” judged the study tool’s content validity. By conducting pilot research with 10% of the study sample (*n* = 14), who were chosen randomly from the same environment and by previous inclusion criteria, face validity was examined. The students who took part in the pilot study were not allowed to engage in the larger study. The necessary changes were made, some questions were added, others clarified, and some were removed based on the data that was gathered.

Study Implementation Process.

I. Pre-intervention Phase:

### (A) Initial preliminary assessment

Initial data was gathered about the participants’ socio-demographic characteristics, their knowledge, adaptive healthy measures, and attitudes toward PCOS before the implementation of the health education intervention. A brief introduction to the goals, processes, voluntary nature of participation, confidentiality and anonymity declarations, and questionnaire cover page were all included. Participants were made aware that they could withdraw from the study at any moment, there would be no negative repercussions, and they could choose to have any case data acquired for the study destroyed. The informed consent form must be signed by the participants and submitted back by ordinary mail to participate.

### (B) Content development of health education regarding PCOS

The developed health education was created based on the participants’ preliminary assessment results and according to recommendations from the [31]. It consisted of seven interactive modules (4 h sessions) in Arabic that covered the following topics: “overview of PCOS, etiology and risk factors, signs and symptoms, diagnosis, complications, preventive measures, and treatment of PCOS.” It aimed at increasing health awareness regarding PCOS, encouraging and promoting adaptive healthy measures to prevent PCOS, and promoting positive attitudes regarding PCOS.

### (C) Website development of health education regarding PCOS

The development team gathered to discuss how each module would be prepared and formatted, as well as how the modules’ sessions would be broken down. The program was designed with defined goals, pertinent materials, and instructional techniques. In addition, it was developed to provide easy access to web-based education that incorporates sources to increase knowledge, support healthy measures, and foster positive attitudes regarding PCOS. This is done using a set of technical and instructional standards developed by a web-based design team, and an information technology expert. The researchers provided the web designer with word-processed documents representing each distinct learning session, videos, a storyboard of suggested graphics, and a description of interactions.

The development team met once a week to go over any issues that might occur, to offer ideas and comments where appropriate, and to address any queries that might have come up while each session was being developed. The web-based design team and researchers chose a learning platform that gave users access to data, tools, and resources to support the administration and delivery of education about PCOS and associated preventive actions online. To get rid of Wix’s pop-up advertisements, the researchers bought a distinctive website domain URL.

The site map and links to the tutorials, forums, and other resources are all accessible from the main screen. The web-based module also included a chat room where students and students conducting research could post questions and engage in conversation. To get user feedback on the learning experience and improve the interface, post-learning discussions and comments were created. Additionally, each student’s frequency and amount of time spent were noted.

To make it simple for the user to choose another menu, the primary menu is also continuously shown on the upper bar. No session could be skipped because the navigation was sequential from session 1 to session 7. The intervention group participants could move on to the following screen, replay the current screen, or go back to the previous screen at any time during a session. They were given as much time as they needed to consider the data displayed on each screen.

The researchers developed the content of the web-based health education sessions. Besides, they drew lines between the elements to indicate how they were linked together. These lines helped dictate sessions’ organization, navigation, and hyperlinks. Moreover, it was arranged into seven sessions. It was displayed by using text, pictures, and videos.

The content was uploaded to the website by the researchers and professional programmers (https://healthconcerns.tech/). The link to the website was distributed to a panel of five external reviewers (experts in community health nursing, women’s health and midwifery nursing, and professional programmers) to systematically test the content’s validity, clarity, consistency, accuracy, applicability, and format before the application of the web-based education. Any specific guidelines or suggestions from the experts’ evaluation were recorded and considered while creating the web-based teaching sessions. The traditional training session that was given to the control group’s students’ material was the same.

The face validity of the web-based modules was assessed concurrently with the review process using a sample group of students (*n* = 14) who had no prior knowledge of PCOS to assess the clarity, applicability, and reliability of the module and to determine the approximate time needed to implement these sessions. Each student in the intervention group completed a module each week and gave the content producer detailed comments afterward. In most cases, students’ input noted areas that needed clarification and more examples, as well as those that were unclear and confusing. During the review process, student feedback was considered. By clicking on the link, participants could view the questions and submit their responses.

### Intervention phase

#### The intervention condition

The research website was recommended to the students in the intervention group of the study who had completed the pre-self-administered questionnaire. There, they learned more about the study and what participating in it entailed. The members of the intervention group were encouraged to create accounts on the website using personal passwords after logging in. An informational session was conducted to teach the intervention group’s members how to utilize the learning management system (LMS) before they started their virtual education. The participants were given instructions to complete the module on their own for the duration of the session after receiving a brief orientation to the navigational features in addition to the help screen in the module. The module can be used on mobile devices, laptops, and personal computers. The online education was only given to the intervention group.

The intervention group’s participants were permitted to access the module as many times as necessary to finish all the sessions (https://healthconcerns.tech/). The participants were instructed to finish at least one and no more than two modules per week. Each module took 30 to 60 min to complete. The intervention was therefore planned to be finished in roughly 5 to 7 weeks. They were also told to heed the health advice that was given. Through website chat, all the participants’ inquiries were addressed, and they received feedback.

#### The control condition

Regarding the traditional health education group, the students had full access to traditional face-to-face health education regarding PCOS for a similar duration and by the same researchers. They were divided into seven groups. Each group had nine to ten students. The nursing faculty’s instructional hall served as the location for the sessions. Each group’s session lasted 5 h over 2 days per week (10 a.m.–12 p.m.). Lectures, discussions, brainstorming, blackboards, printed handouts, and audiovisual materials were employed as teaching and learning tools throughout the sessions.

### Post-intervention phase

Participants’ knowledge, adaptive healthy measures, and attitudes about PCOS were evaluated using the same pre-test questionnaire for the intervention and control groups. Additionally, feedback about the website was assessed once after completion of the module for the intervention group. All participants from the intervention and control groups started and completed the PCOS educational module. The control group was invited and granted access to the same web-based instructional module once data collection, evaluation, and analysis were completed.

### Ethical considerations

#### Ethical approval

was obtained from the Faculty of Nursing Research Ethics Committee (IRB No.P.0360). Informed consent was obtained from all participants in the study and their participation was entirely voluntary and non-for-profit. The research conforms to the provisions of the Declaration of Helsinki (as revised in Brazil in 2013). All participants gave informed consent for the research, and their anonymity was preserved. Administrative permission was obtained from the Faculty of Nursing for data collection. After explaining the purpose of the study and guaranteeing data confidentiality, we were able to secure both written and verbal informed consent from the participants. ClinicalTrials.gov Identifier: NCT06192381. Additionally, the participants were made aware of their ability to leave the study at any time without providing a reason. Using a secure computer and internet connection, all study data was entered into a specific, password-protected electronic database. The predetermined study team members could only access the computerized database. To ensure a trail of data entry into the database, routine automated backups were performed. The chief researcher oversaw and was responsible for keeping all records obtained from the participants during the study in a secure environment.

### Statistical analysis

The statistical package for social sciences (SPSS) software, version 23 (Armonk, NY: IBM Corp.), was used to enter and analyze the data. The analysis was conducted according to the intention-to-treat principle. The Kolmogorov-Smirnov test was utilized to evaluate the data’s normality, and descriptive statistics in the form of frequencies and percentages were used to illustrate the results. For continuous variables, the arithmetic mean, and standard deviation were employed, whereas percentages were used for categorical variables. A separate t-test was utilized to compare the differences in the effectiveness of the intervention program between and within the intervention and control groups. To determine whether there was a positive or negative correlation between the research variables, Pearson correlation coefficients were used. At *p* < 0.05, statistical significance was established.

## Results

Table (1) portrays that the mean age of the studied students was 19.3 ± 0.7 and 19.2 ± 1.1 years for traditional health education and web-based health education groups, respectively. About one-third (33.8%) and more than one-quarter (27.9%) were in the 2nd academic grade for traditional and web-based health education groups respectively. About residence, 77.9% and 72.1% of traditional and web-based health education groups, respectively, lived in rural areas. In addition, less than one quarter 14.7% of the traditional group, and 19.1% of the web-based group had a positive family history of PCOS. Regarding the family history of PCOS, the two groups did not vary substantially from one another (*P* = 0.493).

About menstrual health history, 67.6% and 57.4% of traditional and web-based health education groups, respectively, reported that the number of menstrual cycles in the last year was 3–5 days. Most students in the traditional and web-based health education groups (88.2%, and 80.9%, respectively) had usual bleeding.

Concerning students’ BMI, 50% of the traditional group, and 52.9% of the web-based group had normal body weight. The mean BMI was 24.3 ± 5.0 and 23.8 ± 4.9 for the traditional group and web-based group, respectively. The two groups did not differ significantly in terms of their BMI (*P* = 0.890).

Regarding the source of PCOS knowledge, less than half of the traditional group (44.2%), and 42.9% of the web-based group, their main source of information about PCOS was family members. In addition, 34.9% of the traditional group and 42.9% of the web-based group reported that their source of information was the healthcare team, respectively. The two groups did not differ significantly in terms of their sources of information (*P* = 0.996).

Table 2 shows that before the intervention, the knowledge levels of 70.6% and 73.5% of the students in the traditional and web-based groups, respectively, were poor (*P* = 0.702). However, following the health education intervention, 2.9% and 32.4% of the students in the traditional and web-based groups, respectively, demonstrated a good level of knowledge. (*P* < 0.001).

Pre-health education intervention results for students’ adaptive healthy measures of PCOS showed that 17.6% and 20.6% of the students in the traditional and web-based groups had satisfactory results (*P* = 0.830). However, after receiving health education, 19.1% and 42.6% of students in the traditional and web-based groups, respectively, demonstrated satisfactory adaptive healthy measures (*P* < 0.001).

Regarding the attitudes of students concerning PCOS, 11.8% and 16.2% of students in the traditional and web-based groups, respectively, demonstrated favorable attitudes toward PCOS pre-health education intervention (*P* = 0.458). However, after receiving health education, students in the traditional and web-based groups displayed favorable attitudes in 30.9% and 57.4% of the cases, respectively (*P* = 0.002).

**Table 1:** Participants’ socio-demographic characteristics at the baseline assessment, menstrual history, and source of PCOS knowledge between the traditional and the web-based groups (*N* = 136)

Variables	Traditional health education group		Web-based health education group		Significance	
	*N* = 68	%	*N* = 68	%		
**Age (years)**						
18–19	48	70.6	42	61.8	*P* = 0.277	
20–21	20	29.4	26	38.2		
x̄±SD	19.3 ± 0.7		19.2 ± 1.1		t = 0.557	*P* = 0.578
**Academic year**						
1st	23	33.8	19	27.9	*P* = 0.672	
2nd	22	32.4	21	30.9		
3rd	11	16.2	13	19.1		
4th	12	17.6	15	22.1		
**Residence**						
Rural	53	77.9	49	72.1	*P* = 0.428	
Urban	15	22.1	19	27.9		
**Income**						
Enough for the basic needs	32	47.1	23	33.8	*P* = 0.276	
Enough for the basic needs and emergency	26	38.2	31	45.6		
Able to save	10	14.7	14	20.6		
**Presence of a family history of PCOS**	10	14.7	13	19.1	*P* = 0.493	
**Degree of kinship**						
1st	6	60.0	8	61.5	*P* = 0.940	
2nd	4	40.0	5	38.5		
**Age at menarche (years)**						
< 13	21	30.9	24	35.3	*P* = 0.857	
13–15	37	54.4	35	51.5		
> 15	10	14.7	9	13.2		
**Number of menstruation days in the last year**						
< 3 days	4	5.9	7	10.3	*P* = 0.408	
3–5 days	46	67.6	39	57.4		
> 5 days	18	26.5	22	32.4		
**The interval between menstrual cycle**						
< 1 month	12	17.6	18	26.5	*P* = 0.215	
1 month or above	56	82.4	50	73.5		
**Amount of menstrual bleeding**						
Mild bleeding	8	11.8	13	19.1	*P* = 0.235	
Usual bleeding	60	88.2	55	80.9		
**Body Mass Index (BMI)**						
Underweight	9	13.2	11	16.2	*P* = 0.890	
Normal	34	50	36	52.9		
Overweight	16	23.5	13	19.1		
Obese	9	13.2	8	11.8		
**Source of knowledge about PCOS**						
Family members	30	44.2	29	42.9	*P* = 0.996	
Friends	5	7	4	6.1		
Healthcare professionals	24	34.8	25	36.7		
Mass media	9	14	10	14.3		

**Table 2:** Comparison of the total score and level of knowledge, adaptive healthy measures, and attitudes between the two groups pre- and post-intervention (*N* = 136)

Variables	Traditional health education group		Web-based health education group		Significance
	*N* = 68	%	*N* = 68	%	
**Knowledge (pre-intervention)**					
Poor	48	70.6	50	73.5	*P* = 0.702
Fair	20	29.4	18	26.5	
**Knowledge (post-intervention)**					
Poor	50	73.5	21	30.9	*P* = < 0.001
Fair	16	23.5	25	36.8	
Good	2	2.9	22	32.4	
**Adaptive healthy measures (pre-intervention)**					
Unsatisfactory adaptive healthy measures	55	80.9	54	79.4	*P* = 0.830
Satisfactory adaptive healthy measures	13	17.6	14	20.6	
**Adaptive healthy measures (post-intervention**)					
Unsatisfactory adaptive healthy measures	56	82.4	39	57.4	*P* = < 0.001
Satisfactory adaptive healthy measures	12	19.1	29	42.6	
**Attitudes (Pre-intervention**)					
Negative	60	88.2	57	83.8	*P* = 0.458
Positive	8	11.8	11	16.2	
**Attitudes (post-intervention)**					
Negative	47	69.1	29	42.6	*P* = 0.002
Positive	21	30.9	39	57.4	

*P* Significance. * Significant (*p* < 0.05)

Table (3) presents a comparison of the traditional and web-based health education groups according to their total knowledge, adaptive healthy measures, and attitude scores pre and post-intervention. Concerning the total students’ knowledge score post-intervention, the difference in knowledge between the traditional 22.1 ± 10.2 and web-based group 32.2 ± 10.5 was highly significant. Regarding the adaptive healthy measures score, there was a highly significant improvement in comparison to the traditional group 6.5 ± 2.9 to the web-based group 8.9 ± 2.4. This difference was highly significant. As regards total students’ attitudes regarding PCOS, there was a highly significant improvement in comparing the score of the traditional group 11.7 ± 5.2 to the web-based health education group 18.2 ± 4.9.

**Table 3:** Mean difference between Total Knowledge, Adaptive Healthy Measures, and Attitudes Scores between the Two Groups Pre- and Post-Intervention (*N* = 136)

Variables	Traditional health education group *N* = 68	Web-based health education group *N* = 68	t(*P*)
	Mean ± SD	Mean ± SD	
Pre-intervention			
Total knowledge score	20.6 ± 10.2	20.5 ± 9.8	0.933 (0.353)
Total adaptive healthy measures score	5.9 ± 2.8	6.2 ± 3.0	0.603 (0.548)
Total attitudes score	9.9 ± 4.8	10.5 ± 5.2	0.699 (0.486)
**Post-intervention**			
Total knowledge score	22.1 ± 10.2	32.2 ± 10.5	6.540 (< 0.001)*
Total adaptive healthy measures score	6.5 ± 2.9	8.9 ± 2.4	5.229 (< 0.001)*
Total attitudes score	11.7 ± 5.2	18.2 ± 4.9	7.502 (< 0.001)*

t: paired sample t-test *P*: Significance. * Significant (*p* < 0.05)

Table (4) illustrates the correlation between total knowledge, adaptive healthy measures, and attitudes of students’ pre- and post-web-based sessions. Weak positive correlations were observed in the traditional group between the total score of students’ knowledge, adaptive healthy measures, and attitudes toward PCOS post-intervention. However, in the web-based group, weak positive correlations were observed between the total score of students’ knowledge, adaptive healthy measures, and attitudes toward PCOS post-intervention.

**Table 4:** Correlation between total knowledge, adaptive healthy measures, and attitudes scores between two groups pre- and post-intervention

Variables	Traditional health education group (*N* = 68)											
	Pre-intervention						Post-intervention					
	Total knowledge score		Total practice score		Total attitudes score		Total knowledge score		Total practice score		Total attitudes score	
	**r**	***P***	**r**	***P***	**r**	***P***	**r**	***P***	**r**	***P***	**r**	***P***
**Total knowledge score**			0.246	0.043	0.250	0.040			0.605	0.011	0.311	0.010
**Total adaptive healthy measures score**	0.246	0.043			0.259	0.033	0.605	0.011			0.280	0.021
**Total attitudes score**	0.250	0.040	0.259	0.033			0.311	0.010	0.280	0.021		
**Variables**	**Web-based health education group** (*N* = 68)											
	**Pre-intervention**						**Post-intervention**					
	**Total knowledge score**		**Total practice score**		**Total attitudes score**		**Total knowledge score**		**Total practice score**		**Total attitudes score**	
	**r**	***P***	**r**	***P***	**r**	***P***	**r**	***P***	**r**	***P***	**r**	***P***
**Total knowledge score**			0.244	0.045	0.246	0.043			0.279	0.021	0.256	0.035
**Total adaptive healthy measures score**	0.244	0.045			0.273	0.024	0.279	0.021			0.328	0.032
**Total attitudes score**	0.246	0.043	0.273	0.024			0.256	0.035	0.328	0.032		

r: Pearsons’ Correlation. *P*: Significance. * Significant (*p* < 0.05)

Table (5) illustrates the module usefulness domain, and 79.4% of the students strongly agreed that the module subject was needed in their field, and they could recommend taking this module to a friend with a total mean of 14.852 ± 0.796, indicating that strongly agree prevailed among 92.9% of the student’s responses. Regarding the objectives and learning materials domain, 83.8% of the students strongly agreed that the module objectives were clear, and 76.5% of them strongly agreed that the learning and teaching methods encouraged participation and active learning, with a total mean of 25.926 ± 1.33, indicating that strongly agree prevailed among 92.5% of students’ responses.

Concerning the web-based module vs. face-to-face class domain, 70.6% of them strongly agreed that the time they spent online would have been better spent in a face-to-face class and the ability to review online presentations multiple times, with a total mean of 14.691 ± 0.777, which indicated that 92% of the student’s responses in this domain strongly agreed.

Regarding the usability and accessibility domain, 82.4% of the students strongly agreed that the links provided were still activated and operated. The mean of this domain was 14.220 ± 1.157, indicating that 89.4% of the students strongly agreed. The total mean of the six domains was 95.308 ± 2.395 which indicated that 91.7% of the students’ responses in this domain strongly agreed.

**Table 5:** Students’ feedback of the designed web-based learning about the usefulness of the module, objectives and learning materials, design and structure

Criteria	Strongly agree		Agree	
	*N* = 68	%	*N* = 68	%
The usefulness of the module x̄±SD: 14.852 ± 0.796				
The module subject is important in your field	51	75.0	17	25.0
The module subject is needed in your field	54	79.4	14	20.6
You are satisfied with this module	35	51.5	33	48.5
You can recommend taking this module to a friend	54	79.4	14	20.6
Objectives and learning materials x̄±SD: 25.926 ± 1.33				
The module objectives are clear	57	83.8	11	16.2
Learning materials are well-organized, easily understood	50	73.5	18	26.5
The teaching sessions were well prepared and presented in a logical order.	50	73.5	18	26.5
Recorded slides helped in understanding the material in the module	33	48.5	35	51.5
Thelearningandteaching methods encourage participationandactive learning	52	76.5	16	23.5
The module covers different learning styles (visual, auditory, verbal, and kinesthetic)	44	64.7	24	35.3
Learningmaterialsare providedfor download	49	72.1	19	27.9
Design and structure x̄±SD: 14.705 ± 0.978				
Themodulefollowsaconsistent and organized structure so that navigation does not change from one session to another.	50	73.5	18	26.5
The module is running smoothly (easy to use)	50	73.5	18	26.5
The module presentation style is attractive	36	52.9	32	47.1
The font styles and sizes are consistent	48	70.6	20	29.4
Interaction and assessment x̄±SD: 10.911 ± 0.859				
The module offered ample opportunities for interaction (discussion board, chatting, e-mail) between students and instructor	38	55.9	30	44.1
Sharing and discussion forum in the module worked well	40	58.8	28	41.2
Quizzes were a good way to evaluate my understanding of the learning material	52	76.5	16	23.5
We-based module vs. face-to-face class x̄±SD: 14.691 ± 0.777				
Web-based modules are more convenient than face-to-face class	43	63.2	25	36.8
The time you spent online would have been better spent in a face-to-face class	48	70.6	20	29.4
You can feel comfortable with technology tools	44	64.7	24	35.3
Ability to review online presentations multiple times	48	70.6	20	29.4
Usability and Accessibility x̄±SD: 14.220 ± 1.157				
The module is accessed easily, for free	28	41.2	40	58.8
The module can be found easily on the main site’s homepage	32	47.1	36	52.9
The links provided are still activated, operated	56	82.4	12	17.6
The website or server is accessible all the time	35	51.5	33	48.5
Total x̄±SD: 95.308 ± 2.395				

## Discussion

Web-based health education has shown positive effects in enhancing students’ knowledge and skill performance as well as boosting their self-efficacy in performing nursing skills. Students have expressed a high level of satisfaction with this approach [32]. The current generation of young adults is one of the most frequent users of digital technologies, including social media, mobile phones, and wireless information-sharing platforms [33]. Our study offers new insights into the beneficial effects of web-based health education on students’ knowledge, health behavior, and attitudes.

The findings of the current study on knowledge acquisition revealed an improvement in the total score of knowledge related to PCOS in the traditional health education group post-intervention, with a mean of 22.1 ± 10.2 and 20.6 ± 10.2, respectively. This complex topic is effectively simplified in web-based health education using visuals, animations, high-quality images, and videos.

Online health education provides students with fresh and engaging learning opportunities. According to a study by Aniket [34], health education successfully increased awareness and changed subjective perceptions of PCOS. Similar studies have shown an improvement in knowledge of PCOS after the intervention. These results also support Sowmya et al. 2013’s claim that there was a noticeable difference in knowledge scores about PCOS among teenage females following a comprehensive teaching program on the condition.

Furthermore, these results are also consistent with those of Colwell [35], who stated that girls affected by PCOS felt more motivated and aware of using preventative health measures after participating in a clinical research study. By learning about the short- and long-term health effects of PCOS, girls were able to experience physical and psychological benefits and interact with their healthcare providers more frequently. Additionally, our findings align with those of Hassan [36], who found that health education interventions enhance women’s awareness. Kang and Seomun [37] also noted that a web-based nursing education program positively impacts the clinical performance and knowledge levels of nurses and nursing students.

It is essential to equip women with accurate information tailored to their specific circumstances, as this increases their involvement in treatment planning and breaks the cycle of misinformation that can lead to negative health outcomes [34]. A significant increase in knowledge levels was observed among students in the intervention group after the implementation of the web-based intervention compared to the control group. Participants in the intervention group, who received web-based education and consultation, reported significantly higher levels of eHealth literacy and knowledge at the 3-month follow-up than those in the control group, as reported in a study by [17].

Moreover, Kathryn and Kathryn [38] support the results, stating that at immediate and long-term follow-up, the intervention group had better knowledge of HPV after the web-based intervention and more positive attitudes toward HPV vaccination than the control group. However, these results differ from those of Mehrdad [39], who discovered that web-based learning is as effective as conventional health education and leads to comparable achievement and course satisfaction. The results of the present study also differ from those of Farley [40], who found that web-based health education is inferior to traditional lectures in terms of educational value and enjoyment. Traditional health education is more effective than web-based lessons in the learning process.

The results of the current study show a highly significant difference in health behavior related to PCOS between the web-based health education group and the traditional health education group post-intervention (*P* < 0.001). This difference can be explained by the students recognizing the importance of PCOS-related management and the success achieved through web-based health education during the follow-up period. These results indicate that implementing the web-based intervention and improving students’ knowledge led to increased adherence to recommended healthy measures that could enhance the understanding of diet, physical exercise and healthy lifestyles. Similarly, Guvenc [17] found that teenagers in the intervention group (web-based education and consultation) reported significantly higher levels of healthy behaviors at the 3-month mark compared to those in the control group.

Moreover, these results are consistent with those of Sahar [41] who demonstrated that an internet-based obesity intervention program could be a valuable tool for promoting healthy eating and exercise habits among adolescents with obesity. Additionally, Bashirian [42] concluded that utilizing web-based interventions is an effective educational strategy for preventing smoking among adolescent girls. Furthermore, a study by Pischke [43] indicated that participation in a web-based intervention was linked to higher odds of reduced alcohol and cannabis use among students enrolled in the intervention compared to the control group. These findings contrast with those reported by Stöhr [44], who found no statistically significant differences in performance between traditional and online students before and after the intervention.

A well-designed web-based educational application can have a positive impact on student attitudes toward web-supported instruction and the Internet [45]. Web-based education helps overcome some barriers associated with face-to-face education and offers greater flexibility for learning [42]. Learners’ attitudes play a crucial role in achieving desired learning outcomes. A positive outlook influences learning effectiveness, motivation, application of knowledge, and overall learning outcomes [46].

In the current study, students’ attitudes toward the web-based health education course on PCOS were positive. They found it convenient, engaging, simple to navigate, and helpful. The students also felt that the activities in web-based learning enhanced their learning experience and enabled them to learn at their own pace. Furthermore, compared to the control group, students in the intervention group showed significant improvements in their attitudes following the implementation of the web-based intervention. Educational messages were informative, negative perceptions about PCOS decreased, and supportive beliefs increased.

Similarly, Bashirian [42] found that, after a web-based intervention, the experimental group’s mean score of favorable attitudes toward smoking significantly decreased compared to the control group. Furthermore, the findings of studies by Fathi [47], are congruent with the results of the present study. Additionally, these findings align with those of Eman [29], who demonstrated that web-based health education is an excellent source of information to assist overweight and obese individuals in making dietary and exercise changes for weight management.

Overall, web-based health education was successfully implemented, improving the nursing students’ knowledge of PCOS. Those who utilized the module reported adopting healthier habits compared to those who did not. Additionally, students expressed positive attitudes towards PCOS. The benefits of online health education include convenience, ease of access, and self-paced learning. No harm or unintended effects from the trial were observed. On the other hand, there are drawbacks to web-based health education, including the time instructors must spend building and developing it, teaching skills, filming videos, maintaining, and upgrading the program, and dealing with uploading issues. However, overall, the benefits outweigh the drawbacks as the same outcomes were discovered by [48].

## Conclusion

According to the study’s findings, both traditional and web-based health education interventions improved participants’ knowledge, health practices, and attitudes toward PCOS. However, the web-based health education intervention was more successful than traditional health education. This may be due to the interactive features of the module, which include videos and vibrant visuals that were well-received by students. They found it practical, adaptable, and convenient. However, further study is needed with a larger and more diverse sample from various geographic locations.

### Recommendations

Based on the study’s results and conclusions, the following suggestions are proposed: conduct a community-based dissemination campaign to promote the developed web-based health education module for PCOS. Additionally, integrate web-based health education into all health promotion programs to encourage healthy behavior.

### Limitations of the study

The limitations of the study included the inability to validate participants’ self-reported healthy measures. Additionally, some students in web-based learning may require improved self-discipline and computer skills. The current study did not directly observe the health behavior of students. Future studies should aim to validate the self-reported responses. The generalization of the findings is limited, as only nursing students from one faculty were examined.

### Relevance for clinical practice

Health education is an essential task for healthcare professionals. Web-based strategies could enhance results, improving students’ knowledge, health behaviors, and attitudes regarding PCOS because they are more effective and interactive for students. Web-based procedures should be considered for incorporation in all health promotion programs to enhance healthy behavior.

### Electronic supplementary material

Below is the link to the electronic supplementary material.

Supplementary Material 1

## Data Availability

The data that support the findings of this study are available from the corresponding author upon reasonable request.
