# Supplementary material for: Effect of web-based health education on nursing students’ knowledge, adaptive healthy measures and attitudes regarding polycystic ovary syndrome: a randomized controlled trial
Source: BMC Nurs. 2024 Jul 15;23:479. doi: 10.1186/s12912-024-02015-7 (PMC11247781; doi:10.1186/s12912-024-02015-7)
Supplement: Supplementary file 1 — Supplementary Material 1 [file 12912_2024_2015_MOESM1_ESM.pdf]

# A questionnaire to measure the information, practices and attitudes of female students University with polycystic ovary syndrome

* Indicates required question

Personal data

1. Age*

2. Study group:*

3. Mother's education level: *

*Mark only one oval.*

- He does not read and he does not write
- Reads and writes
- primary education
- Preparatory education
- high school education
- intermediate education
- university
- after university

4. Mother's job *

*Mark only one oval.*

- Working
- Unskilled manual laborer
- Businesses
- Semi-professional
- Professional

5. Economic situation *

*Mark only one oval.*

- mortgagor
- Enough basic expenses
- Sufficient for basic expenses and emergencies
- Able to save

6. Residence *

*Mark only one oval.*

- urban
- rural

## Health history

7. Is there anyone in your family who suffers from polycystic ovary syndrome? *

*Mark only one oval.*

- Yes *Skip to question 8*
- No *Skip to question 9*

8. Do the following: Keep track of what you're looking for*

9. Weight (kg)*

10. Length (cm)*

11. Date of first menstrual cycle *

*Mark only one oval.*

- Less than 3 years .
- 15 years
- More than 15 years

12. Number of days of the menstrual cycle *

*Mark only one oval.*

- Less than 3 days
- 5 days
- More than 5 days

13. The time interval between menstrual cycles *

*Mark only one oval.*

- Less than one month
- One month
- More than 2 months

14. Menstrual bleeding*

*Mark only one oval.*

- Normal bleeding (you need a changing absorbent pad in 3-4 hours)
- No bleeding
- Mild bleeding (spots of blood)
- Severe bleeding (you need to change a high suction pad in 1-2 hours)

## Questionnaire elements

15. Definition of ovaries *

*Mark only one oval.*

- Glands that produce hormones that regulate metabolism and growth, tissue function, sexual function, sleep, and mood.
- A pair of long narrow ducts located in the female's abdominal cavity that transport male sperm cells to the eggs.
- I don't know.

16. Number of ovaries: *

______________________________

17. Ovarian function *

*Check all that apply.*

- [ ] Eggs contain DNA.
- [ ] Production of hormones (estrogen and progesterone).
- [ ] Moving the egg from the ovary, where it is produced, to the central canal of the uterus.
- [ ] Feeding and sheltering the fertilized egg and embryo.
- [ ] I don't know.
- [ ] Other

18. If you choose another, mention the function of the ovaries:

_______________________________

19. Definition of polycystic ovary syndrome *

*Mark only one oval.*

- ( ) It is a common hormonal disorder among women between 15-45 years old. It appears in about 10% of women of reproductive age. *Skip to question 21*
- ( ) I don't know. *Skip to question 21*
- ( ) others...

20. If you choose another, mention the definition of polycystic ovary syndrome:

_______________________________
_______________________________
_______________________________
_______________________________
_______________________________

21. Causes of polycystic ovary syndrome *

*Check all that apply.*

- [ ] Hormonal imbalance in the body.
- [ ] Increased cell resistance to insulin.
- [ ] Genetics.
- [ ] Inflammation in the body.

22. Risk factors *

*Check all that apply.*

- [ ] Obesity
- [ ] Family history
- [ ] Insulin resistance
- [ ] High level of androgen hormone.
- [ ] I don't know.

23. Symptoms accompanying the disease *

*Check all that apply.*

- [ ] Irregular or absent menstruation.
- [ ] menorrhagia
- [ ] Increased hair growth throughout the body.
- [ ] acne
- [ ] Hair growth in male areas such as the beard and chest.
- [ ] hair loss.
- [ ] Sleep disorders.
- [ ] Weight gain.
- [ ] Mood disorders, such as depression, anxiety, and eating disorders.
- [ ] No Symptom.
- [ ] I don't Know.
- [ ] Other

24. If you choose another, mention the symptoms accompanying the disease.

_______________________________

25. Diagnosis *

*Check all that apply.*

- [ ] Clinical examination and observation of symptoms.
- [ ] Lab tests: Blood tests to look for signs of additional male hormones.
- [ ] Pelvic ultrasound to measure ovarian size and signs of ovarian cysts
- [ ] Other: _______________________________

26. Complications *

*Check all that apply.*

- [ ] Endometrial cancer.
- [ ] Abnormal bleeding in the uterus.
- [ ] Type 2 diabetes/insulin resistance.
- [ ] Hypertension
- [ ] Cardiovascular diseases
- [ ] Spontaneous miscarriage or premature birth.
- [ ] brain attack
- [ ] Non-alcoholic fatty liver disease.
- [ ] I don't know
- [ ] others

27. If you choose another, mention the complications.

_______________________________

## Methods of prevention and treatment

28. Nutrition *

*Check all that apply.*

- [ ] Limit sugar and preservatives in food.
- [ ] Limit your intake of foods high in carbohydrates and fats.
- [ ] Eat fruits, vegetables, whole grains and legumes.
- [ ] Eat foods that contain protein instead of carbohydrates that worsen insulin sensitivity.
- [ ] Eat foods that contain anti-inflammatories such as olive oil, tomatoes, and leafy greens.
- [ ] Eat iron-rich foods such as spinach, eggs, and broccoli.
- [ ] Avoid caffeine consumption.
- [ ] Take vitamins and supplements such as vitamin B- A, zinc, cinnamon, turmeric, and ginger.

29. Lose weight*

*Check all that apply.*

- [ ] Follow a special diet to lose excess weight.
- [ ] Exercise regularly.
- [ ] Taking medications to lose excess weight.

30. Staying away from wrong healthy habits*

*Check all that apply.*

- [ ] Avoid smoking and passive smoking.
- [ ] Reducing mental and psychological stress, relaxation and psychological comfort.
- [ ] Get enough sleep hours day and night.

31. Pharmaceutical treatment and natural treatment*

*Check all that apply.*

- [ ] Take pills to regulate hormones in the body.
- [ ] Eat herbs such as ginseng, basil, and licorice root.

32. Periodic follow-up*

*Check all that apply.*

- [ ] Periodic and continuous follow-up of diabetes.
- [ ] Pay attention to changes in the menstrual cycle

33. Sources of information about polycystic ovary syndrome:*

*Check all that apply.*

- [ ] Family members
- [ ] Friends
- [ ] Health team
- [ ] Media
- [ ] I don't know..

34. Some daily practices*

*Mark only one oval per row.*

| | Yes | No |
|---|---|---|
| Do you avoid reducing your intake of sugar and preservatives in food?ا | | |
| Do you limit your intake of foods high in carbohydrates and fats? | | |
| Do you avoid eating too much fast food that contains preservatives? | | |
| Do you avoid excessive consumption of soft drinks or stimulants (tea, coffee)? | | |
| Do you eat a lot of fruits, vegetables, whole grains, legumes, and foods rich in fiber? | | |
| Do you eat foods that contain protein instead of carbohyd | | |

Do you eat foods that contain anti-inflammatory drugs, such as olive oil, tomatoes, and leafy vegetables?

Do you eat foods rich in iron, such as spinach, eggs, and broccoli?

Do you take vitamins and nutritional supplements such as B-A, zinc, cinnamon, turmeric, and ginger ?

Do you exercise regularly every day for at least 30 minutes?

Do you follow a special diet to lose excess weight?

Do you get enough sleep (6-8 hours) daily?

Do you avoid exposure to psychological and nervous stress?

Do you perform regular and continuous examinations to detect polycystic ovary syndrome early?

35. Attitudes of female university students regarding polycystic ovary syndrome

*Mark only one oval per row.*

| | Agree | Neutral | Disagree |
|---|---|---|---|
| Is it a disease that women suffer from for life? | | | |
| It is a hereditary disease that cannot be avoided | | | |
| It is a disease that leads to serious physical and psychological problems | | | |
| It is a disease that cannot be cured or controlled | | | |
| It is a disease that I do not feel anxious, afraid of, or disturbed by at all | | | |
| I believe that an infected woman will never give birth | | | |
| I have a satisfactory and optimistic outlook toward | | | |
| Delay in diagnosing and treating the disease leads to multiple serious problems | | | |

Regular examinations and examinations are very important for early detection of the disease

Changing lifestyle is one of the most important ways to prevent and treat disease

I believe that eating healthy food and reducing excess weight are among the most important ways to prevent and treat disease

.

There is nothing I can do to avoid getting sick

The disease does not negatively affect the social life of women

If I suffer from illness, I should not inform others about my illness

This content is neither created nor endorsed by Google.

Google Forms
